# Thulium fiber vs. holmium: YAG lasers in urology: insights from the FDA MAUDE database

**DOI:** 10.1007/s00345-025-05919-4

**Published:** 2025-10-04

**Authors:** Juanita Velasquez Ospina, Etienne Gozlan, Adam Williams, Aravindh Rathinam, Archan Khandekar, Jonathan Katz, Robert Marcovich, Hemendra N. Shah

**Affiliations:** Desai Sethi Urology Institute, University of Miami, Miller School of Medicine, 1120 NW 14th St #2107, 15th Floor, Miami, FL 33136 USA

**Keywords:** Holmium YAG laser, Thulium fiber laser, MAUDE, Adverse event, Urology

## Abstract

**Purpose:**

To compare adverse events (AEs) associated with Thulium Fiber Lasers (TFLs) and Holmium: YAG (Ho: YAG) lasers reported in the FDA MAUDE database, and to examine changes in TFL-related AEs following the FDA’s 2021 Class II recall.

**Methods:**

The FDA MAUDE database was searched for events between 2018 and 2024 using “LUMENIS MOSES,” “LUMENIS VERSAPULSE,” “SOLTIVE,” and “TFL + {YEAR}.” Events were classified as device, patient, staff or environmental, and graded using the Gupta system (Levels I-IV). Exclusions included non-urologic procedures, insufficient detail, or duplicates. Subgroup analyses considered prostate vs. non-prostate, TFL pre- vs. post-recall, and Ho: YAG by pulse modulation (MOSES vs. standard). Chi-square or Fisher’s exact tests were used for categorical data, and Wilcoxon rank-sum tests for Gupta comparisons.

**Results:**

954 events were included (467 TFL, 487 Ho: YAG). Console malfunctions were more common with Ho: YAG (44.4%, *p* < 0.0001), while fiber breaks more with TFLs (57%, *p* < 0.0001). Patient-involving AEs occurred more with Ho: YAG (35.3%) compared to TFLs (13.5%, *p* < 0.0001). Most events were Level I: 83.9% TFL vs. 59.3% Ho: YAG (*p* < 0.0001). Level II events were higher with Ho: YAG (39.8%), and Level III with TFLs (2.1%, *p* = 0.0436); one Level IV event occurred (Ho: YAG group). Post FDA recall, TFL-related Level II and III events decreased significantly. Prostate vs. non-prostate stratification showed no differences. MOSES use in Ho: YAG had fewer fiber issues, but more Level II events compared to standard holmium.

**Conclusion:**

Lasers in urology appear safe, with most AEs being free of patient or staff harm and classified as minor. Post-recall improvements in TFL safety profiles suggest effective corrective action.

## Introduction

Since their invention in the 1960s, lasers have been widely studied and increasingly adopted in medicine—especially in surgery [1, 2]. Their application in urology dates to the 1970s and has become essential in modern practice, currently being used for multiple procedures [3, 4]. Among the first to emerge—and still widely used— is the holmium: yttrium-aluminum-garnet (Ho: YAG) laser, with a 2120 nm wavelength and efficient water absorption, making it highly effective for urological procedures [4, 5]. More recently, the thulium fiber laser (TFL) emerged as a promising alternative, with a 1940 nm wavelength, allowing for greater water absorption and potentially superior stone ablation [5]. Furthermore, recent data suggest higher stone free rates with TFLs [6, 7]. 

Efficacy aside, both are considered safe, but still carry risk of malfunction and adverse events (AEs), requiring ongoing surveillance [8]. Complications reported across different specialties include device malfunction incidents, provider or patient harm and operating room fires [4, 9]. 

The Manufacturer and User Facility Device Experience (MAUDE) database, a publicly accessible tool established by the U.S. Food and Drug Administration (FDA) for post-market surveillance, monitors medical device-related AEs [10]. It contains reports from manufacturers, healthcare facilities and individuals, and has guided safety actions. For example, on August 15, 2021, the FDA issued a Class II recall for the Soltive Premium and Soltive Pro Super Pulsed Laser Systems (TFL based) due to thermal injury during ureteral stone treatment after a user surpassed standard power presets (20 W) [11]. In response, Olympus released a software update adding an 8 W ureteral preset, safety alerts for settings above 20 W, and revised instructions for use.

In 2014, Althunayan et al. analyzed MAUDE-reported Ho: YAG laser-associated AEs in urology [12]. Since then, studies examined device-related complications for urological procedures such as ureteroscopy with lithotripsy (URS), benign prostatic hyperplasia and prostate cancer therapies [4, 13–17]. However, these have focused on specific procedures.

We aimed to characterize and compare AEs associated with TFL and Ho: YAG lasers from the MAUDE database across all urologic procedures. Additionally, we aimed to assess whether the profile of TFL-related AEs changed following the 2021 FDA recall. This information can help guide safer use of laser devices, support clinical decision-making and raise awareness of product limitations.

## Methods

**Fig. 1:**
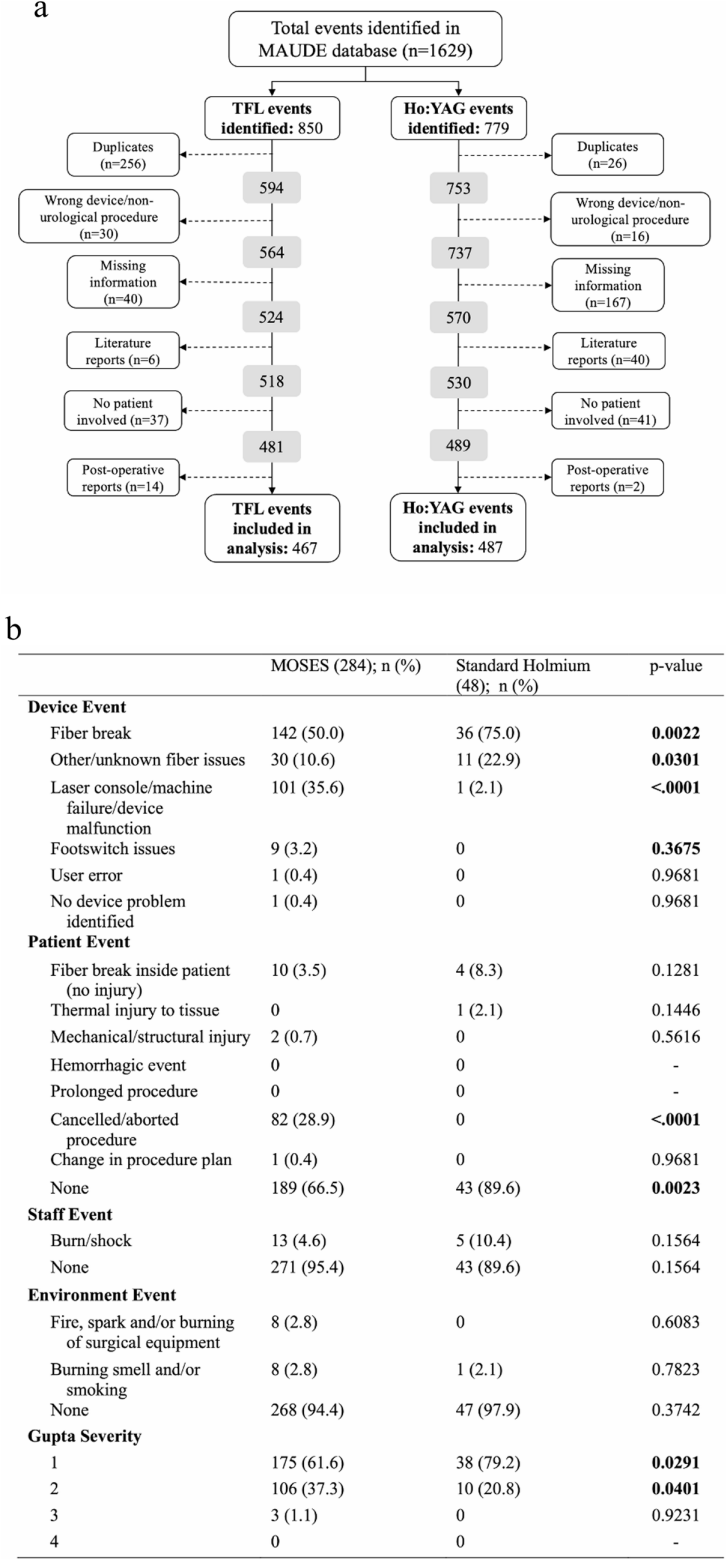
MAUDE database analysis of TFL and Ho: YAG laser adverse events: **a** flowchart of stepwise exclusion of events, **b** Ho: YAG laser adverse events: standard vs. MOSES pulse modulation

In October 2024, the FDAs MAUDE database was queried using the keywords “LUMENIS MOSES,” “LUMENIS VERSAPULSE,” “SOLTIVE,” and “TFL + {YEAR},” with the year ranging from 2018 to 2024. Two databases were created, one per laser. Data obtained included occurrence date, report date, manufacturer, brand name, and event description. Each was reviewed individually and classified into 4 categories: device (machine and components), patient, staff or environmental event, with those involving multiple categories classified accordingly. Furthermore, the events were grouped according to their specific type (Table 1). Each was assigned a grade ranging from I-IV using the Gupta classification system for AEs [18]. Exclusion criteria and numbers are detailed in Fig. 1a. Each AE was classified into a single device category, those without a clearly identified device malfunction were classified as “no device problem identified.”

**Table 1:** Overall and procedure-specific distribution of adverse events (Prostate vs. Non-Prostate)

	Total				Prostate				Non-Prostate		
	TFL (467) n (%)	Ho (487) n (%)	p-value		TFL (6) n (%)	Ho (36) n (%)	p-value		TFL (231) n (%)	Ho (185) n (%)	p-value
Device Event											
Fiber break	266 (57)	186 (38.2)	**< 0.0001**		3 (50)	16 (44.4)	0.8010		160 (69.3)	84 (45.4)	**< 0.0001**
Other/unknown fiber issues	34 (7.3)	49 (10.1)	0.1258		1 (16.7)	2 (5.6)	0.3355		12 (5.2)	27 (14.6)	**0.0019**
Laser machine malfunction	63 (13.5)	216 (44.4)	**< 0.0001**		1 (16.7)	16 (44.4)	0.3740		32 (13.9)	60 (32.4)	**< 0.0001**
Footswitch issues	95 (20.3)	32 (6.6)	**< 0.0001**		1 (16.7)	1 (2.8)	0.2680		19 (8.2)	13 (7.0)	0.7867
User error	2 (0.4)	2 (0.4)	0.9882		0	0	–		1 (0.4)	0	0.9391
Unknown	7 (1.5)	2 (0.4)	0.0783		0	1 (2.8)	0.6931		7 (3.0)	1 (0.5)	0.0811
Patient Event											
Fiber break inside patient (no injury)	32 (6.9)	14 (2.9)	**0.0041**		0	2 (5.6)	0.5572		29 (12.6)	10 (5.4)	**0.0205**
Thermal injury to tissue	3 (0.6)	2 (0.4)	0.661		0	0	–		3 (1.3)	0	0.2574
Mechanical/ structural injury	3 (0.6)	3 (0.6)	0.9789		0	1 (2.8)	0.6931		3 (1.3)	2 (1.1)	0.8531
Hemorrhagic event	1 (0.2)	0 (0)	0.3237		0	0	–		1 (0.4)	0	0.9391
Prolonged procedure	6 (1.3)	3 (0.6)	0.2673		0	0	–		5 (2.2)	1 (0.5)	0.2326
Cancelled/aborted procedure	13 (2.8)	147 (30.2)	**< 0.0001**		0	13 (36.1)	0.1533		11 (4.8)	38 (20.5)	**< 0.0001**
Change in procedure plan	5 (1.1)	3 (0.6)	0.3992		1 (16.7)	2 (5.6)	0.3355		4 (1.7)	1 (0.5)	0.3873
None	404 (86.5)	315 (64.7)	**< 0.0001**		5 (83.3)	18 (50.0)	0.1973		175 (75.7)	133 (71.9)	0.4347
Staff Event											
Burn/shock	17 (3.6)	26 (5.3)	0.2041		1 (16.7)	3 (8.3)	0.4737		9 (3.9)	12 (6.5)	0.3301
None	450 (96.4)	461 (94.7)	0.2041		5 (83.3)	33 (91.7)	0.4737		222 (96.1)	173 (93.5)	0.3301
Environment event											
Fire, spark and/or burning surgical equipment	112 (24)	12 (2.5)	**< 0.0001**		0	1 (2.8)	0.6931		67 (29)	2 (1.1)	**< 0.0001**
Burning smell and/or smoking	29 (6.2)	17 (3.5)	0.0518		1 (16.7)	0	0.1429		15 (6.5)	5 (2.7)	0.1175
None	326 (69.8)	458 (94)	**< 0.0001**		5 (83.3)	35 (97.2)	0.2683		149 (64.5)	178 (96.2)	**< 0.0001**
Gupta Severity											
1	392 (83.9)	289 (59.3)	**< 0.0001**		4 (66.7)	16 (44.4)	0.4004		170 (73.6)	120 (64.9)	0.0691
2	65 (13.9)	194 (39.8)	**< 0.0001**		1 (16.7)	19 (52.8)	0.1870		52 (22.5)	62 (33.5)	**0.0169**
3	10 (2.1)	3 (0.6)	**0.0436**		1 (16.7)	0	0.1439		9 (3.9)	3 (1.6)	0.2404
4	0 (0)	1 (0.2)	0.3338		0	1 (2.8)	0.6931		0	0	–

Ho = Ho: YAG

For subgroup analyses, events were classified as prostate and non-prostate. The unspecified organ cases were not included in the subgroup analysis. TFL cases were labeled pre- or post–FDA recall, and Ho: YAG cases as MOSES, standard Holmium, or unclear (according to pulse-modulation settings). Statistical analysis was performed using SAS v9.4. Chi-Square tests or Fisher’s exact tests were performed on the categorical data. Fisher’s exact tests were applied in instances where cell counts were insufficient to meet the assumptions of the Chi-square test. A Wilcoxon rank-sum test was used to compare the Gupta Scores. A p-value of < 0.05 was considered statistically significant.

## Results

Of 1629 events identified, 850 (52.2%) were TFLs and 779 (47.8%) Ho: YAG lasers. After exclusions (383 TFL, 292 Ho: YAG), 467 and 487 events remained, respectively.

Device issues were identified in most cases; only 1.5% (TFL) and 0.4% (Ho: YAG) lacked an identifiable problem. Laser device/console malfunction (including malfunction of parts, errors, shutdown, overheating) were significantly more common with Ho: YAG lasers (*p* < 0.001), while fiber breaks more with TFLs (*p* < 0.0001).

Patient events were significantly more common with Ho: YAG lasers (35.3%) than TFLs (13.5%) (*p* < 0.0001), with most AEs classified as Gupta Level I (minor, no intervention required), a total of 59.3% and 85% in the Ho: YAG and TFL groups, respectively. Level I events were significantly more frequent in the TFL group while Level II more in the Ho: YAG group. TFLs had significantly more Level III events (*n* = 10, 2.1%) and there was only one Level IV event overall in the Ho: YAG group, a bladder perforation with subsequent patient death.

### Prostate and non-prostate procedures

Prostate procedures comprised 7.4% of Ho: YAG events and 1.3% of TFLs, while non-prostate were 38% and 49.5%, respectively (Table 1). No significant differences were seen in prostate cases. In non-prostate, fiber breaks overall and those inside patients were significantly more common with TFLs, cancelled/aborted procedures were more common with Ho: YAG and regarding severity, only Level II events were significantly higher with Ho: YAG.

### Pulse modulation settings

Compared with standard Ho: YAG, MOSES was associated with fewer fiber breaks (50.0% vs. 75.0%), but significantly more procedure cancellations (28.9% vs. 0%) (Fig. 1b) While most AEs in both groups were Level I, these were more common with standard Ho: YAG (79.2% vs. 61.6%), whereas Level II events were more frequent with MOSES (37.3% vs. 20.8%).

### TFL events before and after the FDA recall

**Fig. 2:**
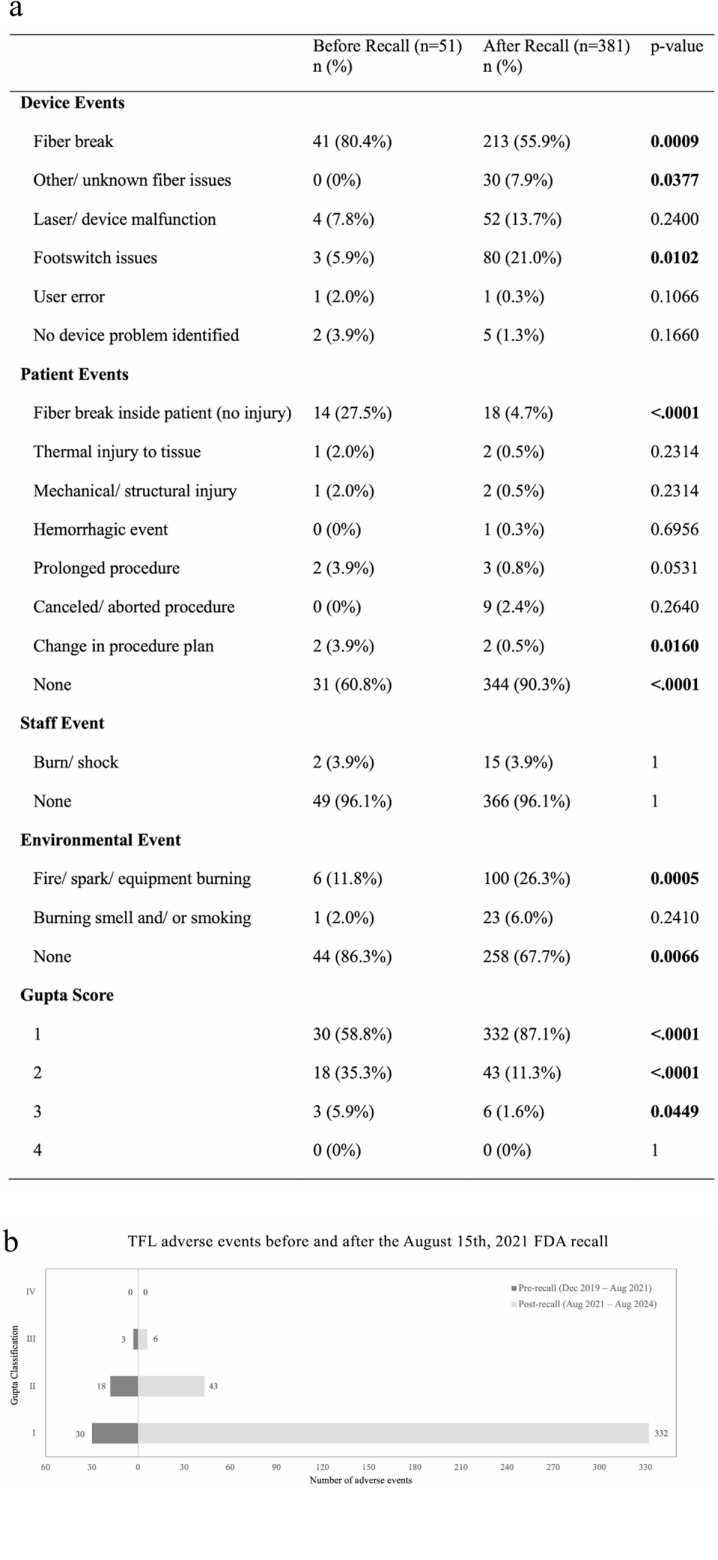
TFL adverse events before and after the August 15, 2021 FDA recall: **a** summary table of event types and frequencies, **b** graphical representation of comparative distribution

Of 467 TFL-related events, 432 were eligible for the recall subgroup analysis (51 pre-, 381 post-recall) (Fig. 2a). Overall, a significantly higher proportion of events were free of patient harm after the recall (60.8% pre vs. 90.3% post). Fiber breakages inside patients decreased significantly, along with Level II and III events (Fig. 2b).

## Discussion

Recently published studies have analyzed MAUDE-reported AEs but have focused on specific procedures or diagnoses [13, 16, 17]. Ours is the first comparing MAUDE-reported AEs between TFLs and Ho: YAG lasers across all urologic procedures. We found most AEs did not involve patient events (86.5% in TFL, 64.7% in Ho: YAG), and TFLs were associated with fewer patient-involved events (*p* < 0.0001).

Most Ho: YAG laser device-related events were device or laser fiber malfunctions (44.4%), followed by fiber breaks (38.2%). A 2014 MAUDE-based study similarly found 46% of Ho: YAG-related AEs involving fiber breakage or device failure, although more recently Juliebø-Jones et al. reported an even higher rate (78.8%) of fiber breakage [12, 13]. However, neither study included TFLs, which we found had significantly higher fiber breakage rates both overall (57%) and in non-prostate procedures. Consequently, there were also significantly more breakages inside patients with TFLs. This may be related to their thinner diameter compared to Ho: YAG, though altogether these results show that laser fibers—especially TFLs—are fragile and careful handling could mitigate risk [5]. Additionally, differences in generator power output may contribute to the observed variation in fiber-related events.

Ho: YAG lasers had significantly more Level II events, most being cancelled or aborted procedures (147/194) which, compared to another study analyzing MAUDE Ho: YAG events, our rate was notably higher (30.2% vs. 5.2%) [13]. This discrepancy could be explained by their focus on lithotripsy procedures, while we included all laser-related procedures. Conversely, their study reported a higher incidence of prolonged anesthesia (14.7%) compared to ours (0.6%), possibly due to more complex cases in their sample or differing definitions. Prolonged anesthesia may reflect equipment troubleshooting, bringing in backup machines or even retrieving fiber pieces. Aborted or changed procedures might also indicate inappropriate treatment indications and could be underreported or not explicitly stated.

Level III events were significantly more common in TFLs, although the number was small (10 AEs; 2.1%). These included two ureteral perforations (one from a fiber break), one renal perforation, one case of intrarenal bleeding, four patients requiring additional procedures (two resulting from fiber breakage), one procedure reportedly prolonged for 7 h due to malfunction of the machine, and one patient whose approach was changed due to an unknown machine error. Ho: YAG laser Level III AEs included one additional procedure and an intrarenal injury (both secondary to fiber breaks), and a ureteral perforation without an identified device event.

Our findings align with Chandramohan et al.’s study reporting a significantly higher rate of mucosal injury with TFL use (28.8%) compared to Ho: YAG lasers (11.1%) during URS [19]. In contrast, a systematic review reported significantly lower rates of intraoperative complications with TFLs (OR 0.34), and a meta-analysis also found a lower incidence when compared to Ho: YAG lasers, though without statistical significance [20, 21]. Similarly, a randomized controlled trial reported fewer intraoperative complications with TFLs during URS [22]. However, most published data focus on a single procedure, whereas the MAUDE database—and consequently our study—covers a broader range of procedures.

Still, most events were Level I (83.9% TFL; 52.3% Ho: YAG), comparable to studies assessing device safety in urology. These have reported Level I rates ranging from 39.4% to as high as 99.3%, depending on devices and procedures included [15–17]. Others using the Clavien–Dindo classification to report outcomes have similarly found 82.5% of complications pertaining to grades I or II (mild) [14]. 

Stratification by procedure demonstrated no significant differences between lasers in prostate cases, which was somewhat expected given the small sample. In contrast, non-prostate procedures accounted for most reports, with events generally classified as Level I (73.6% TFL, 64.9% Ho: YAG). Within Ho: YAG lasers, stratification by pulse modulation suggested that MOSES may reduce fiber-related issues but was more often associated with console malfunctions and cancelled procedures compared to standard Ho: YAG. Most events in both groups were minor, though MOSES had relatively more Level II AEs. However, interpretation is limited by the relatively small number of standard Ho: YAG reports.

Additionally, after the August 2021 FDA TFL-device recall, a significantly greater proportion of events had no patient involvement (60.8% pre-recall vs. 90.3% post-recall), with a significant decrease in Level II and III events [11]. While Level III events were still reported post-recall (3 pre vs. 6 post), the overall reduction and increase in reports without patient harm suggest that the corrective actions had a positive impact, highlighting the importance of ongoing surveillance and adherence to laser safety protocols.

A critical principle in laser use is not surpassing the 43° C thermal threshold, as this can lead to tissue damage [23]. In vitro studies measuring intraureteral fluid have shown high temperatures with laser use, one even reporting higher maximum temperatures with TFLs than Ho: YAG lasers [24]. Gupta et al. recently measured intrarenal temperatures during endoscopic procedures (20 patients), revealing both Ho: YAG and TFLs quickly reached high temperatures at high-power settings, though TFLs reached significantly higher maximum temperatures during “pop-dusting.” [25] In addition to intrarenal fluid temperature, factors such as thermal safety distance (thermal dose + laser fiber tip distance) and exposure duration also contribute to thermal injury risk [26]. Strategies including ureteral access sheaths, continuous irrigation, and intermittent laser activation can help mitigate these effects [26, 27]. 

We found no statistically significant differences in thermal or structural tissue injuries; however, there were three reports of thermal injury with TFLs, while the two reported with Ho: YAG lasers were skin burns. We also found two ureteral and one intrarenal perforation in the TFL group, compared to one ureteral, one intrarenal, and one bladder perforation in the Ho: YAG group. The bladder perforation— the only Level IV AE— occurred during a holmium laser enucleation of the prostate. Reportedly there was gas explosion with bladder rupture requiring conversion to open surgery; one hour later, the patient’s blood pressure dropped, acute myocardial infarction was diagnosed, and the patient ultimately died.

In addition to intraoperative complications, long-term issues such as ureteral strictures have been reported, with Para et al. noting a significantly higher incidence following TFL use compared to Ho: YAG during URS (11/238 vs. 4/240, respectively) [28]. In a separate study, Villani et al. compared manufacturer-recommended versus individualized TFL presets and reported a significantly higher stricture rate with the former (11% vs. 1%) [29]. Although we didn’t specifically analyze this complication, several reports in our MAUDE dataset mentioned stricture-related events. However, because of limited procedure details, lack of follow-up, and delayed nature of stricture development, these events could not be reliably assessed or linked to intraoperative laser use. Still, their presence suggests the potential for thermal injury even when using strategies like power settings adjustment or continuous irrigation [23, 24, 28]. Further research is needed to better characterize these complications.

Our study has limitations. The MAUDE database depends on voluntary reporting and is subject to reporting bias. The total number of cases is unknown, preventing incidence calculation, and specific details such as procedure type, laser settings (frequency, power, pulse modulation) and use of adjunctive measures are often missing. Follow-up information is rarely provided, making it difficult to establish causality, and postoperative outcomes such as fever, stone-free rates, and strictures are not reliably captured, limiting assessment of delayed complications. Despite this, we believe our study offers an insight into laser AEs and their overall safety in urology.

## Conclusion

Although lasers are considered safe for urologic use, they can present AEs of varying degrees. Our study describes the types and severity of user-reported events, reflecting experiences that are different from clinically controlled settings. This offers insight into everyday practice and can serve as reference for the kinds of events encountered.

## Data Availability

The data analyzed in this study are publicly available in the U.S. Food and Drug Administration’s Manufacturer and User Facility Device Experience (MAUDE) database (https://www.accessdata.fda.gov/scripts/cdrh/cfdocs/cfmaude/search.cfm).
